# Elevated expression of Tie1 is accompanied by acquisition of cancer stemness properties in colorectal cancer

**DOI:** 10.1002/cam4.1072

**Published:** 2017-05-02

**Authors:** Miku Torigata, Daishi Yamakawa, Nobuyuki Takakura

**Affiliations:** ^1^ Department of Signal TransductionResearch Institute for Microbial DiseasesOsaka UniversitySuitaOsaka565‐0871Japan

**Keywords:** Cancer stem cell marker, cancer stem cells, colorectal cancer, Lgr5, Tie1

## Abstract

The Tie receptors 1 and 2 (Tie1/2) play crucial roles in embryonic angiogenesis. Recent studies suggest enhanced expression of Tie1 in several types of cancer and negative correlations between Tie1 levels and clinical outcome. These observations suggest important functions of Tie1 not only for vascular formation but also in tumorigenesis. Ligands for Tie2, that is angiopoietins 1‐4, have been identified, but not for Tie1. To determine the molecular functions of Tie1, its detailed characterization in tumors would be helpful. Herein, we report that Tie1 is up‐regulated in colorectal cancer. Detailed analysis using tumor‐bearing models and immunohistochemistry combined with Flow cytometric analysis and cell sorting (FACS) revealed that Tie1 protein was expressed in a small population of malignant tumor cells. Intriguingly, Tie1 expression was observed and could be maintained only in vivo. Further analysis using sphere‐formation culture revealed that Tie1‐positive cells are enriched within the population of tumor cells with cancer stemness properties. Indeed, Tie1‐positive tumor cells derived from a murine model overexpressed Lgr5, a typical stemness marker for colorectal cancer. Our results provide a novel insight into Tie1 function in tumorigenesis and suggest clinical applications to target cancer stem cells.

## Introduction

“Tyrosine kinase with immunoglobulin and epidermal growth factor homology domains‐1″ (Tie1) has been identified as a receptor tyrosine kinase‐like orphan (i.e., ligand‐less) receptor 1. Tie1 and its homolog Tie2 are both important for embryonic angiogenesis and vascular integrity 2, 3. Tie1 can heterodimerize with Tie2 and modulates angiogenic processes through angiopoietin (Ang) proteins, a family of vascular growth factors 4. Ang/Tie‐signaling is essential for vessel remodeling and maturation processes. Indeed, constitutive deletion of Tie1, Tie2, or Ang‐1 results in embryonic lethality caused by defects in the vasculature 5, 6, 7. In contrast, although Tie1 shows high homology with Tie2 protein, only the latter interacts with angiopoietin family members Ang‐1, Ang‐2, Ang‐3, and Ang‐4, suggesting complicated regulatory mechanisms to transduce Ang‐mediated signals 8, 9, 10. As suggested by some characteristic differences between Tie1 and Tie2, several papers have reported that Tie1 has specific functions in pathogenesis, such as in pathological adult vasculature and tumor angiogenesis 11, 12.

Previous studies have suggested tumorigenic functions for Tie1 other than in angiogenesis, because it is expressed in various epithelial cancers, such as breast cancer 13, 14 gastric cancer 15, and thyroid cancer 16. In these studies, Tie1 expression was observed in cancer cells themselves, as confirmed by immunohistochemistry. Moreover, these investigations suggested that the corresponding normal tissue did not express Tie1. In clinical practice, Tie1 expression shows a negative correlation with 5‐year survival rates in gastric cancer patients 15. In melanoma cell lines, Tie1 protein levels are positively correlated with the degree of malignant behavior of each cell line 17. Furthermore, in breast cancer, Tie1 was commonly overexpressed in tumors and is found as truncated forms of the intracellular domain, containing the tyrosine kinase catalytic domain 13, 14. This observation indicates that Tie1 possesses pivotal functions for intrinsic signals of tumor cells and thus that modification of the Tie1‐mediated signals might be implicated in tumor progression. Despite the accumulating evidence of Tie1 expression in tumors, its physiological relevance remains to be elucidated.

Here, to obtain insights into Tie1 functions in tumorigenesis, we explored Tie1 expression patterns in different types of cancer. We report that colorectal cancer is Tie1‐positive, albeit expressing this molecule in only a small population of malignant tumor cells in vivo and not apparent under standard in vitro conditions at all. We found that Tie1 expression was associated with cancer stemness properties of the cells. We further determined that Tie1‐positive tumor cells could be cultured in vitro using sphere formation, a condition enriching cancer stem cell (CSC) populations. Our results suggest a functional link between Tie1 and CSC properties and the possibility for applications in CSC studies.

## Methods

### Animals

KSL/Slc nude mice (7–8 weeks of age) were purchased from Japan SLC (Shizuoka, Japan). Mice were housed in environmentally controlled rooms of the animal experimentation facility approved by the Animal Care Committee of Osaka University (Osaka, Japan). All animal experiments were approved by the Animal Research Committee of Osaka University. All experiments were carried out under the guidelines of Osaka University Committee for animal and recombinant DNA experiments.

### Cell culture

Cell lines HT29, HCT116, SW837 (human colon cancers), Colon‐26 (mouse colon cancer), PC3 (human prostate cancer), LLC (mouse lung cancer), and B16 (mouse melanoma) were cultured in RPMI‐1640 medium (Sigma‐Aldrich, St. Louis, MO) supplemented with 10% FBS (Equitech‐Bio, Kerrville, TX) and 1% penicillin/streptomycin (Invitrogen, Carlsbad, CA). TS1 (tumor stromal cell) was cultured in Dulbecco's modified Eagle's medium (Sigma‐Aldrich) supplemented with 10% FBS and 1% penicillin/streptomycin 18. All cell lines other than TS1, which was established in our previous study, were purchased from ATCC (Manassas, VA).

### Transfection and G418 selection

To construct a pEGFP‐N1 stably expressing cell line, HT29 human colorectal cancer cells were plated at a density of 1 × 10^6^ cells per 35 mm dish. After overnight culture, an expression plasmid for EGFP was transfected using Lipofectamine 2000 (Invitrogen). After 24 h, G418 (1 mg/mL) was added to select and enrich for transfected cells. To construct mTie1‐EGFP or EGFP‐expressing cell lines (Colon26‐EGFP cells or Colon26‐mTie1 cells), Colon‐26 mouse colorectal cancer cells were transfected with each expression plasmid as described previously 18.

### In vivo tumor cell xenograft model

HT29 tumor cells (5 × 10^6^ per mouse in 0.1 mL PBS) and HCT116 or SW837, or Colon‐26 (2 × 10^6^ per mouse in 0.1 mL PBS) were inoculated subcutaneously into KSL/Slc nude mice (7–8 weeks of age). Tumor was dissected 21–28 days after implantation. HT29‐EGFP cells were transplanted to the KSL/Slc nude mouse (7–8 weeks of age), and tumors were dissected after 3–4 weeks. Only the cells which were expressing GFP were sorted and sampled as confirmed HT29 cells.

Colon26‐EGFP cells and Colon26‐mTie1 cells were transplanted subcutaneously into KSL/Slc nude mice (7–8 weeks of age), and tumor volume was measured every 3 days from day 5 after transplantation. In this experiment, before transplantation into mice, Colon‐26 cells were transfected with mTie1 expression plasmid as described above. The Tie1‐expressing Colon‐26 cells were then collected by FACS and precultured to expand them; those enriched for mTie1 expression (Fig. S1) were transplanted into mice to assess tumorigenicity.

### Cell proliferation assay

Cell proliferation was determined using the WST‐8 reagent (Dojindo, Kumamoto, Japan) following the manufacturer's instructions. This assay is basically similar to the 3‐[4,5‐dimethylthiazol‐2‐yl]‐2,5‐diphenyl‐tetrazolium bromide (MTT) assay.

### Quantitative reverse‐transcription polymerase chain reaction

Total RNA was isolated using RNeasy Mini Kits (Qiagen, Hilden, Germany). RNA was reverse‐transcribed using the ExScript RT Reagent Kit (Takara, Kyoto, Japan). qRT‐PCR was performed using SYBR Premix Ex Taqll (Takara, Kyoto, Japan) on an Mx3000 QPCR system (Stratagene, La Jolla, CA). Levels of the specifically amplified cDNAs were normalized to the level of glyceraldehyde 3‐phosphate dehydrogenase (GAPDH), a housekeeping gene. We used the following primer sets: 5′‐CTG CCT GCA ATC TAC AAG GT‐3′ and 5′‐CCC TTG GGA ATG TAT GTC AGA‐3′ for human Lgr5, and 5′‐AGG AGA CCA AGG CCC GTT A‐3′ and 5′‐ATC AGT TGG GCC TCC AGA GA‐3′ for human CK20, and 5′‐TGA TGA CAT CAA GAA GGT GGT GAA G‐3′ and 5′‐ TCC TTG GAG GCC ATG TGG GCC AT‐3′ for human GAPDH, respectively 19, 20, 21.

### Flow cytometric analysis and cell sorting

Single‐cell suspensions from tumors were prepared using a standard protocol 21. Flow cytometric analysis was performed using a FACSCalibur (BD Pharmingen, Franklin Lakes, NJ), and cell sorting was performed using a FACSAria (Becton Dickinson, Franklin Lakes, NJ). The antibodies used were phycoerythrin (PE)‐conjugated anti‐human Tie1 monoclonal antibody (mAb), PE‐conjugated goat IgG (R&D systems, Minneapolis, MN), FITC‐conjugated anti‐mouse CD31 mAb (BD Pharmingen), FITC‐conjugated anti‐mouse CD45 mAb and APC‐conjugated anti‐mouse CD45 mAb (eBioscience, San Diego, CA). The PE‐high cell population and PE‐negative population were sorted, respectively.

### Immunohistochemistry

Immunostaining was performed on 10 *μ*m cryostat sections of mouse tumor tissue as described previously 22. Each section was washed with ice‐cold PBS for 10 min and fixed with 4% PFA/PBS for 15 min. For immunohistochemistry, rat anti‐mouse CD31 mAb (BD Pharmingen), rabbit anti‐Tie1 H‐180 antibody (Santa Cruz Biotechnology, Santa Cruz, CA), biotin‐conjugated anti‐mouse CD45 mAb (BD Pharmingen), and rat anti‐PDGFR‐*β* mAb 18 were used as primary antibodies. Alexa Fluor 546 goat anti‐rabbit IgG (Invitrogen), Alexa Fluor 488 goat anti‐rat IgG (Invitrogen), Streptavidin‐Fluorescein Isothiocyanate (BD Pharmingen) were used as the secondary antibodies.

### Laser scanning microscopy

Photographs were taken using a confocal microscope TCS/SP5 (Leica). Images were processed using Adobe Photoshop software (Adobe Systems, Mountain View, CA).

### Clinical database analysis

We analyzed Tie1 mRNA expression levels in human cancer samples using ONCOMINE (https://www.oncomine.org/), a cancer database platform 23. P‐values were computed by ONCOMINE software using Student's *t*‐test.

### Culture together with different growth factors or inhibitors

Human colorectal cancer cells were plated at a density of 1 × 10^6^ cells in 6‐well dishes. Cells were grown in RPMI‐1640 medium with 2 ng/mL TGF*β*1, 100 ng/mL EGF, 100 ng/mL bFGF, 100 ng/mL HGF, 50 ng/mL PMA, or DMSO as a control. After 24 h, Tie1 expression was analyzed by FACS. For exposure to inhibitors, cells were cultured as above with 10 *μ*mol/L MG132 (proteasome inhibitor), 100 mol/L Bafilomycin A1 (H ± ATPase inhibitor), 10 *μ*mol/L Brefeldin A (Intracellular protein transport inhibitor), 50 mmol/L NH_4_Cl (endocytosis inhibitor), 1 *μ*mol/L Trichostatin A (Histone deacetylase inhibitor), 20 *μ*mol/L PD98059 (MAP kinase kinase inhibitor), 50 *μ*mol/L LY294002 (Phosphatidylinositol‐3 kinase inhibitor), 10 *μ*mol/L SB203580 (p38 MAP kinase inhibitor), 50 *μ*mol/L JNK Inhibitor II, or DMSO as a control. After 24 h, Tie1 expression was analyzed by flow cytometric analysis as described above.

### Coculture with tumor stromal cells

HT29 human colorectal cancer cells expressing dark red fluorescent protein E2 crimson were cocultured with or without TS1 tumor stromal cells in 6‐well dishes. After 7 days, Tie1 expression was analyzed by FACS.

### Sphere formation

HT29 human colorectal cancer cells were plated at a density of 2 × 10^6^ cells per Ultra‐Low Culture Dish (Corning, One Riverfront Plaza, Corning, NY) for tumor sphere‐formation assays. Cells were grown in RPMI‐1640 medium supplemented with 1 ×  B27 supplement (Invitrogen), 20 ng/mL epidermal growth factor (EGF; Peprotech), 10 ng/mL basic fibroblast growth factor (bFGF; Peprotech) and 1% penicillin/streptomycin. After 7 days, Tie1 expression was analyzed by FACS.

### Drug treatment

HT29‐EGFP tumor cells (5 × 10^6^ per mouse in 0.1 mL PBS) were inoculated subcutaneously into KSL/Slc nude mice (7–8 weeks of age). HT29‐EGFP tumor‐bearing mice were treated with fluorouracil (5‐FU; Kyowa Hakko Kogyo, Tokyo, Japan) or saline (Otsuka Pharmaceutical Factory, Tokushima, Japan) as a control intraperitoneally at a dose of 100 mg/kg b.w. once every 3 days for 9 days.

### Gene set enrichment analysis

To assess whether a Tie‐1‐dependent gene expression signature was significantly enriched in 2D‐ or 3D‐cultured colorectal cancer cells, we performed GSEA using transcriptomic data previously deposited in Gene Expression Omnibus (GEO; https://www.ncbi.nlm.nih.gov/geo/). A list of potential Tie‐1‐responsive genes (i.e., >2‐fold increase upon Tie‐1 knockdown) was obtained comparing Tie‐1 knocked‐down and nontreated HUVECs (GSE27871). Using this gene set, we performed GSEA in preranked mode with default settings for GSE23773, transcriptomic data of HT‐29 colorectal cancer cells cultured in a 3D culture system using NanoCulture plates (NCPs) (Scivax) and in 2D culture.

### Statistical analysis

Data are presented as mean ± standard error of the mean (SEM). Two sets of data were compared using Student's *t*‐test. Values of *P* < 0.05 were considered as statistically significant.

## Results

### Exploration of Tie1 overexpressing human cancers

**Figure 1:**
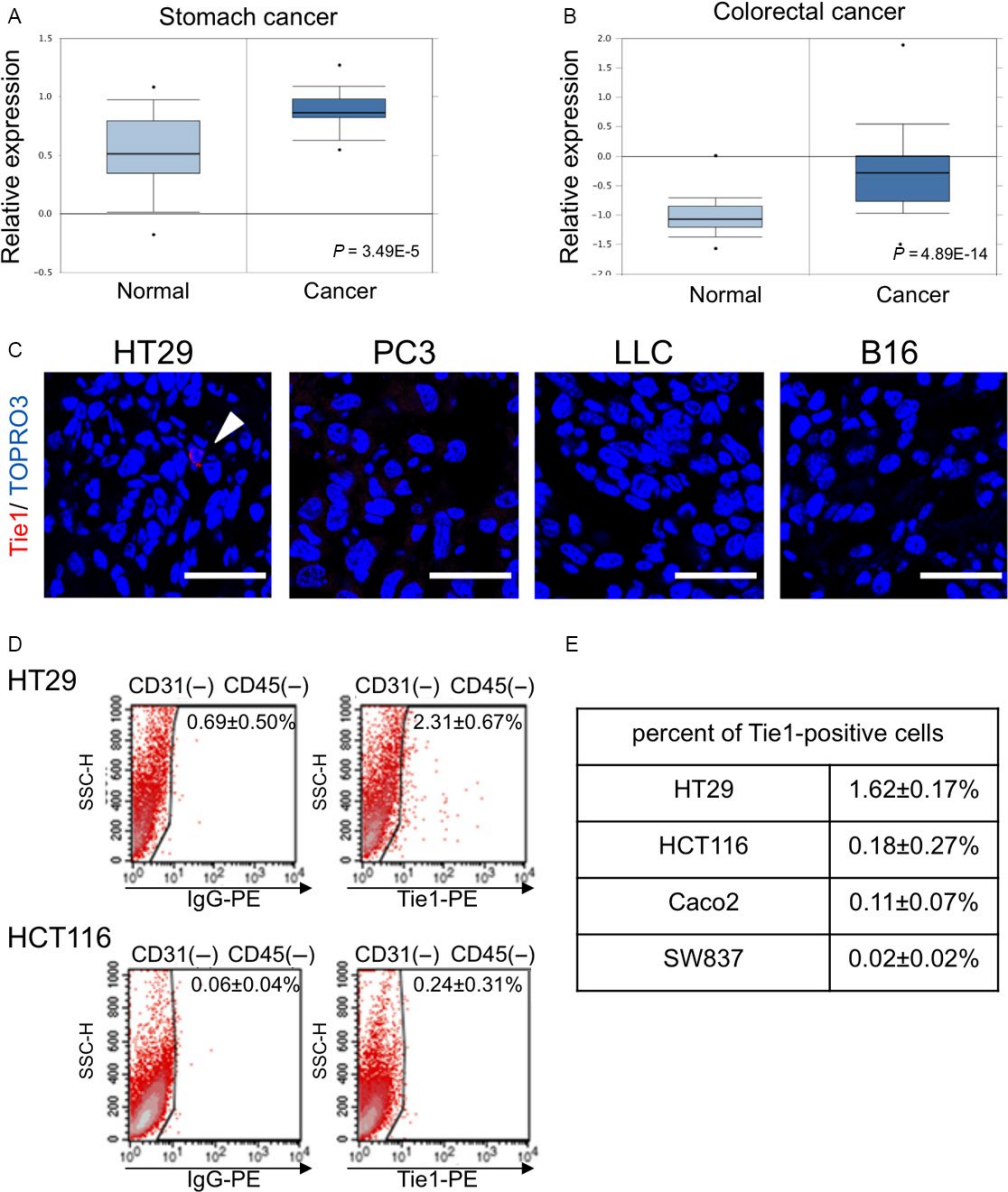
Expression of Tie1 in different tumors. (A, B) Meta‐analysis of Tie1 mRNA expression level in stomach cancer specimens (A) and colorectal cancer specimens (B). Box and whisker plots show enhanced expression of Tie1 in cancer. The relative expression level was visualized for normal samples and cancers from the Oncomine database. Each p‐value was calculated by student's *t*‐test. (C) Representative immunohistochemistry images of Tie1 (red) in the tumors which were derived from different cancer cell lines by subcutaneous inoculation. HT29, human colon cancer cell line; PC3, human prostate cancer cell line; LLC, mouse lung cancer cell line; B16, mouse malignant melanoma cell line. TOPRO3 (blue) was used to counterstain nuclei. (D, E) Flow cytometric analysis of several human colorectal cancer cell lines for Tie1 expression. Representative scatter plots for HT29 and HCT116 cells (D). To eliminate murine endothelial cells which express Tie1, only CD31^−^ CD45^−^ tumor cells from tumor cell suspensions were analyzed. Results are summarized in (E) for HT29, HCT116, Caco2, and SW837 cells.

Elevated expression of Tie1 has been reported in some types of human cancer, such as stomach cancer 24. To obtain insights into associations of Tie1 with tumorigenesis, we first explored Tie1 expression patterns in different types of cancer using Oncomine, a clinical database of human cancer. Consistent with previous reports, a significant elevation of Tie1 expression was observed in stomach cancer (p = 3.49E‐5) (Fig. 1A). In addition, we also found a significant elevation of Tie1 in colorectal cancer (p = 4.89E‐14) (Fig. 1B). This suggests an association of Tie1 with tumorigenic events in intestinal tissue.

### Tie1 is expressed in a fraction of malignant colorectal cancer cells

Using tumor‐bearing mouse models is an effective strategy to examine the properties and associations with surrounding tissues of well‐characterized and/or modified cancer cell lines in vivo. Using C57BL/6 or nude mice bearing subcutaneous tumors, we next performed immunohistochemistry for Tie1 on a number of cell lines derived from human or mouse cancers (HT29, a human colorectal cancer; PC3, a human prostate cancer; LLC, a mouse lung cancer; and B16, a mouse melanoma). Immunohistochemistry of tumors dissected from these mice revealed that Tie1‐positive cells were detected only in the HT29‐bearing mice (Fig. 1C, arrow). To characterize Tie1 expression in this type of cancer in more detail, we analyzed additional human colon cancer cell lines as well as HT29 using cells from tumor‐bearing mice followed by FACS. We observed that Tie1‐positive cancer cells remained after eliminating CD31^+^ endothelial cells and CD45^+^ hematopoietic cells which are known to express Tie1. Intriguingly, although a Tie1‐positive population was essentially absent from HCT116, Caco2, and SW837 cells, a small Tie1‐positive population was again detected in HT29 cells (representative scatter plots; Fig. 1D, summarized in Fig. 1E). The latter cell line is recognized as being relatively more malignant than the other cell lines tested. These observations suggest that Tie1 expression is maintained only in a small population in the tumor, and that this feature might correlate with malignancy.

### Tissue distribution of Tie1‐positive cells using an HT29‐EGFP tumor model

**Figure 2:**
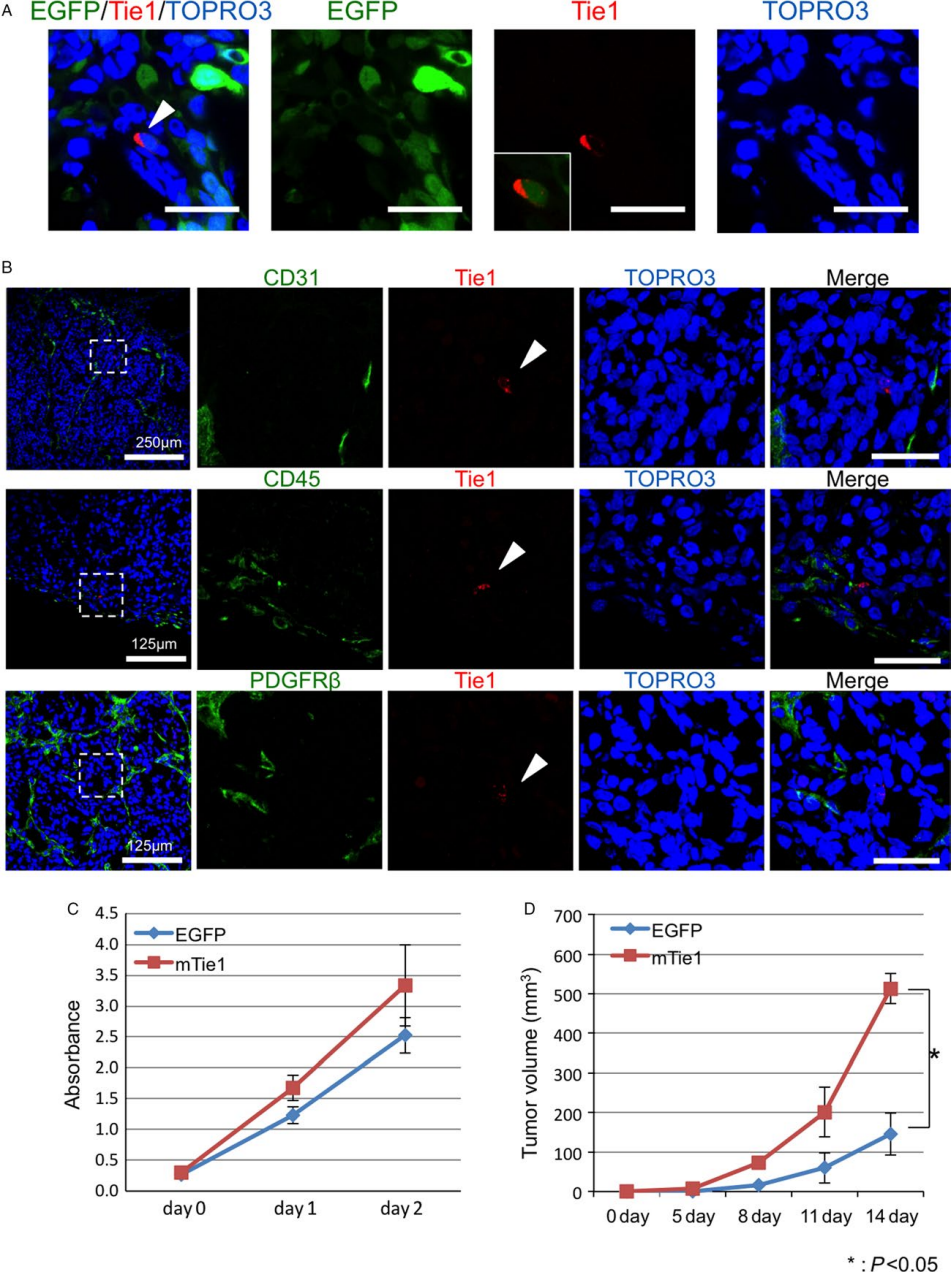
Exploration of the origin of Tie1‐expressing cells in tumors. (A) Immunohistochemistry images of Tie1 (red) in tumor tissues from the mouse subcutaneous EGFP stably expressing HT29 cell implantation model. Representative images showing colocalization of EGFP and Tie1; the arrow shows a Tie1‐positive cancer cell. The inset in the third image shows Tie1‐positive cells merged with EGFP alone. (B) Immunohistochemistry images for different cell markers in the HT29 tumor sections. Note that Tie1 signals (red; arrow heads) do not colocalize with any cell surface markers (green). Top: CD31, vascular endothelial cell marker; middle: CD45, leukocyte marker (here, possibly macrophages); bottom: PDGFR *β*, fibroblast marker. TOPRO3 (blue) was used to visualize nuclei. (C) Growth curve of Colon26‐mTie1 (mTie1) cells and Colon26‐EGFP (EGFP). Data show means ± SD (*n* = 3,*P < 0.05). (d) Quantitative evaluation of tumor volume generated by Colon26‐mTie1 cells (mTie1) or Colon26‐EGFP cells (EGFP) subcutaneously transplanted into nude mice. Data show means ± SD (*n* = 3, *P < 0.05).

To further confirm which type of cell is the origin of the Tie1‐positive cells described above, we constructed an HT29 cell line stably expressing green fluorescent protein (hereafter referred to as HT29‐EGFP). Using this cell line, we performed immunohistochemistry for Tie1 on tumor tissue dissected from the mouse tumor‐bearing mouse. Multiple fluorescence imaging revealed that the Tie1 signals clearly colocalized with EGFP signals (Fig. 2A). To exclude the possibility that Tie1‐positive cells originated from tumor‐associated tissues, we further investigated other typical marker proteins (CD31, CD45, and PDGFR*β* for endothelial cells, white blood cells and fibroblasts, respectively). Because none of these colocalized with the Tie1 signal, we conclude that the Tie1‐positive cells indeed originated from the injected HT29‐EGFP tumor cells, and not from any murine cells (Fig. 2B).

Next, to assess the impact of Tie1 expression on proliferation of cancer cells in vitro and in vivo, we generated colon‐26 cell lines expressing EGFP or mTie1‐EGFP (hereafter referred to as colon26‐EGFP and colon26‐mTie1, respectively) (Fig. S1). There was no significant difference in proliferation rate between colon26‐EGFP and colon26‐mTie1 under in vitro culture conditions (Fig. 2C). In contrast, colon26‐mTie1 cells generated significantly greater tumor volumes in mouse transplantation models in vivo (Fig. 2D). Therefore, we concluded that Tie1 expression is involved in the in vivo growth of cancer.

### Tie1 is not expressed by cancer cell lines maintained in two‐dimensional (2D) cell culture

**Figure 3:**
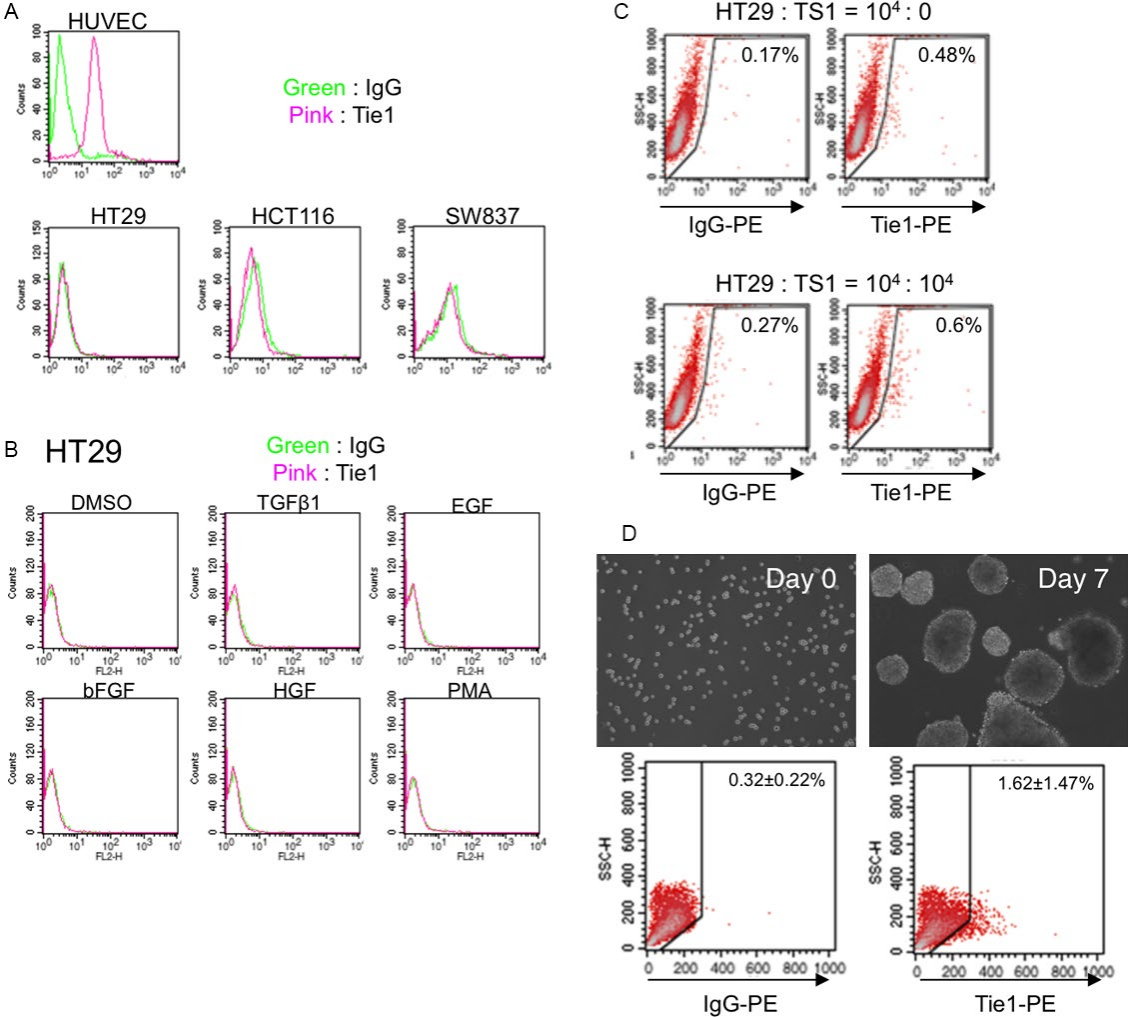
Expression of Tie1 by different tumor cell lines in vitro. (A) Flow cytometric analysis of human colon cancer cell lines HT29, HCT116, and SW837. Tie1 expression under normal culture conditions was analyzed. HUVEC, vascular endothelial cells, were used as a positive control for Tie1 expression. (B) Flow cytometric analysis of HT29 cells after stimulation with different growth factors as indicated. (C) Coculture of cancer stromal cells TS1 with HT2 expressing E2‐crimson, modified version of human colon cancer cell line. After one week coculture, HT29 cells (E2‐crimson‐positive) were analyzed by flow cytometry to confirm Tie1 expression. Cells from culture without TS1 (the two left dot plot data panels) are negative controls. (D) Images show before (Day 0) and after (Day7) sphere formation of HT29 cells. The result of flow cytometric analysis of HT29 cells before and after sphere formation is shown at the bottom of each image.

Having shown that a small population of cancer cells expresses Tie1 in vivo (Fig. 2), we next assessed whether Tie1 expression in cancer cells in normal two‐dimensional cultures (i.e., regular in vitro conditions) can be detected. Despite having detected a small proportion of Tie1‐positive cancer cells in in vivo tumors, Tie1‐positive HT29 cells were essentially absent from in vitro cultures. Tie1 positivity was not observed at all in in vitro‐cultured HCT116 or SW837 cells (Fig. 3A).

Although we could not detect Tie1‐positive HT29 cells under regular in vitro culture conditions, we next investigated whether Tie1 positivity was retained in HT29 cells sorted from tumors developing in mice in vivo. It is noteworthy that Tie1 expression was no longer detectable on HT29 cells after Tie1‐positive cells from tumors in mice were removed and cultured in vitro (Fig.S2). This suggests that Tie1 expression can only be maintained in vivo, and needs to be induced in the specific microenvironment of the tumor.

### Tie1‐expression is enriched in tumor cells with stemness properties

In order to investigate how Tie1 expression is induced, we performed FACS analysis using HT29 cells after stimulation by several different growth factors (TGF*β*1, EGF, bFGF, and HGF). PMA was included as a control stimulator. However, there were no differences in Tie1 expression compared to vehicle control after challenge by any of these factors (Fig. 3B). Next, we cultured HT29 cells with different molecular inhibitors (MG132, Bafilomycin A1, Brefeldin A, NH_4_Cl, Trichostatin A, PD98059, LY294002, SB203580, and JNK Inhibitor II). Here again, there were no differences in Tie1 expression relative to the solvent control (data not shown).

Next, we hypothesized that expression of Tie1 could be induced due to association with tumor stromal cells present in the tumor microenvironment. Thus, we cocultured HT29 cells stably expressing E2‐crimson together with TS1 cells, a tumor‐associated fibroblast cell line which we established and have described in a previous report 18. Coculture for 7 days failed to result in Tie1 induction (Fig. 3C). These results suggest that although Tie1 expression is maintained in the tumor microenvironment, this is not reproduced by exposing HT29 to typical growth factors nor by interaction with cells from a single tumor‐associated lineage.

Next, we focused on correlations of stemness properties with Tie1 expression. To enrich and analyze the cancer stem cell‐like population of HT29 cells, we cultured them on Ultra‐Low Culture Dishes using special culture medium to form spheres (sphere‐formation assay). Sphere formation is a method for detecting cancer stem cells in vitro. FACS analysis revealed that a fraction of the sphere‐forming HT29 cells did express Tie1 in vitro (Fig. 3D).

### Tie1 expression is accompanied by the expression of cancer stemness markers

**Figure 4:**
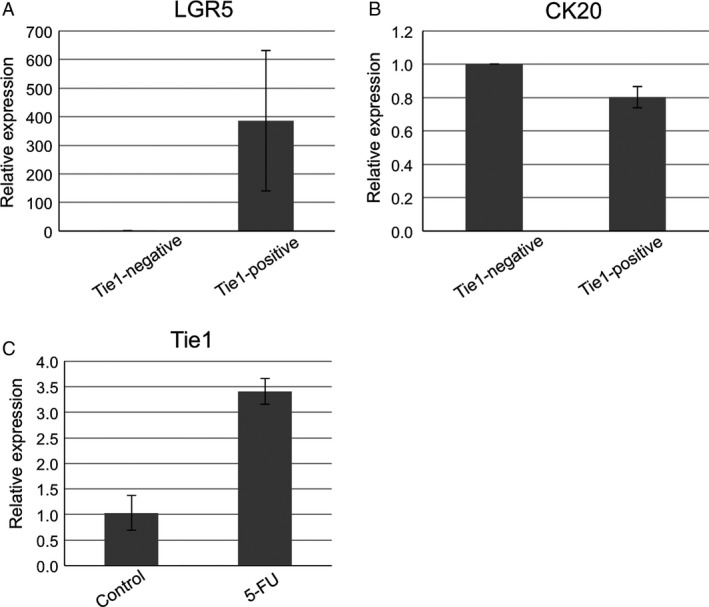
Tie1 expression positively correlates with cancer stem properties in vivo. (A,B) Gene expression analysis of Lgr5, a cancer stem cell marker (A) and CK20, a typical differentiation marker (B) of intestinal tissue. Sorted Tie1‐positive and ‐negative cells recovered from transplanted tumors were analyzed by quantitative RT‐PCR. (C) Gene expression analysis of Tie1 in recovered HT29‐EGFP cells after continuous 5‐FU treatment. Quantitative RT‐PCR analysis was performed on transplanted HT29‐EGFP cells after selection pressure by 5‐FU. Tumor cells were sorted using EGFP signals. Each quantitative RT‐PCR experiment was performed at least three times. Expression levels were normalized to GAPDH, an internal control transcript. Bar represents SD.

Because Tie1‐positive cells were observed in the in vivo tumor and the in vitro sphere‐formation model, we investigated whether Tie1 expression is accompanied by the expression of cancer stem cell marker genes in vivo. Using the HT29 transplantation model, we assessed the expression of Lgr5, CD44, and CD133, major human colorectal cancer stem cell markers 25, and CK20, a characteristic differentiation marker 26 by sorted Tie1‐positive cancer cells. Quantitative RT‐PCR analysis comparing Tie1‐positive and ‐negative cells revealed a slight reduction in CK20 and a significant induction of Lgr5 expression in Tie1‐positive cells. In contrast, expression of CD44 and CD133 was relatively weak in Tie1‐positive cells, and heterogeneous within the LGR5‐high population (i.e., the LGR5‐high population contained cells with different CD44 and CD133 expression levels) (Fig. 4A and B).

It is well known that cancer stem cells show resistance to anticancer drugs. To confirm whether Tie1 expression increased on treatment of HT29 tumor‐bearing mice with anticancer drugs, we assessed its expression by tumor cells collected from mice treated or not treated with the drug 5‐FU. Quantitative RT‐PCR analysis revealed that the expression of Tie1 in 5‐FU‐treated tumor cells was higher than in control cells (Fig. 4C). Taking these data together, we conclude that Tie1 expression is positively correlated with cancer stemness properties of malignant tumor cells.

**Figure 5:**
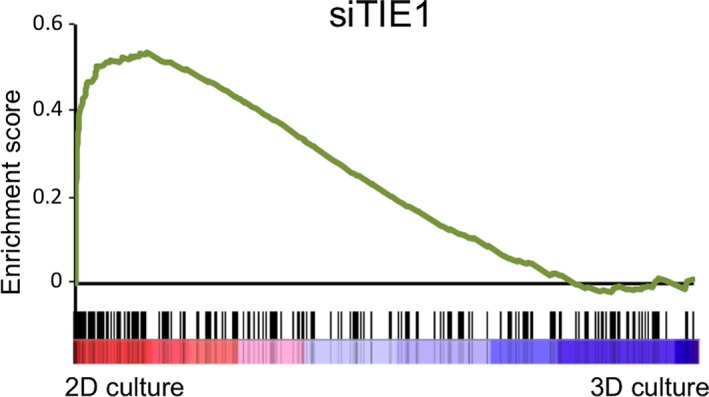
Tie1 deficiency causes cancer cells in 2D‐cultures to commit to nonstem cell‐like cancer cells. GSEA showing that genes highly expressed in Tie1‐deficient cells were up‐regulated under 2D culture conditions in HT29 cells. Genes up‐regulated upon Tie1 knockdown in HUVEC were defined as potential Tie1‐responsive genes. Cells under 3D culture conditions maintain malignant properties such as anchorage‐independent growth. Note that genes predominantly expressed in Tie1‐deficient cells (i.e., genes up‐regulated upon Tie1 knockdown in HUVEC) were highly expressed in 2D‐cultured HT29 cells compared to 3D‐cultured, cancer stem‐like cells (*P* < 0.001).

Transcriptomic data of HT29 cells revealed that potential Tie1‐responsive genes were enriched under 3D‐culture conditions, an in vivo‐imitating technique (Fig. 5). This observation suggests that dysregulation of Tie1 function might have marked effects on many biological signals, which could include tumor progression. Taken together, our findings imply that Tie1 may be of importance as a novel marker of CSCs and could also represent a novel therapeutic target molecule which should be addressed in more detail in further studies.

## Discussion

Although Tie1 and Tie2 have been well characterized in embryonic angiogenesis, recent studies indicate that enhanced expression of Tie1 can be observed in some epithelial tumors, such as breast, stomach, thyroid, and gastric cancer where it is positively associated with poor outcome 13, 14, 15, 16. These observations suggest that Tie1 plays a fundamental role in tumor progression 11. In this study, to elucidate Tie1 expression patterns and to obtain insight into its physiological functions in tumors, we performed immunohistological analyses in a tumor‐bearing mouse model. We identified colorectal cancer as a novel Tie1‐expressing tumor, with Tie1‐positive cells hardly detectable in the normal intestine. Tie1 expression did not influence cancer cell proliferation in regular in vitro cultures, but significantly affected malignant growth of transplanted tumors in vivo (Fig. 2C and D).

Interestingly, we found that only a small population of tumor cells expressed the Tie1 protein and that it was not maintained under normal 2D cell culture conditions. Furthermore, culture of the Tie1‐expressing cell population together with several different growth factors failed to allow retention of Tie expression, suggesting that specific environmental cues in tumors are required for its maintenance. Moreover, coculture of cancer cells with cancer‐associated fibroblasts also failed to maintain Tie1 expression in cancer cells. This suggests that Tie1 expression is likely to be induced by combined effects of several components of the tumor microenvironment.

A series of observations in our studies implied a correlation of Tie1‐positive cells with CSCs, which form a small population and associate with tumor malignancy. The tumor microenvironment including stromal cells and inflammatory immune cells plays an important role in facilitating malignancy and maintenance of CSCs 27, 28. Indeed, we confirmed that Tie1‐positive cells were enriched in the CSC population using sphere‐formation culture conditions in vitro. Tie1‐positive tumor cells highly expressed Lgr5, a stemness marker for colorectal cancer. However, the expression of CD44 and CD133 did not exhibit a pattern similar to LGR5. These results indicate that the LGR5‐positive population was not composed of uniform cells. Moreover, enhancement of cancer stemness properties by exposure to an anticancer drug resulted in increased Tie1 expression in tumor tissue. In addition, we have also shown that Tie1‐positive cells express Bmi‐1, a therapeutic target for colon cancer stem cells (unpublished data)^29^. In a previous study, it was reported that expression of vascular‐related factors, including Tie1, increased in CSCs of human malignant melanoma 17. Our findings in this study extend this finding to other types of cancer.

Tie1 expression (at the mRNA level) was also enhanced in human colorectal cancer (Fig. 1A). This strongly suggests that the phenomena observed in our mouse model might also occur in human clinical cases. However, it is not clear whether Tie1 expression is a cause or result of acquisition of malignancy and/or cancer stemness properties. The increase in Tie1 mRNA in accordance with tumor malignancy in whole tumor tissue (previous reports 24 and Fig. 1B) is not strictly equivalent to the increase in tumor cells expressing Tie1 protein. Thus, it will be important to determine whether the appearance of a very small population of CSCs expressing Tie1 protein can explain the increase in Tie1 at the mRNA level in whole tumor tissue.

Serial transplantation models using small numbers of cancer cells have been employed to determine whether a certain population of cancer cells has the property of CSCs. In our studies, it was difficult to assess the tumorigenic ability of Tie1‐positive HT29 cells sorted from tumor‐bearing mice because of the extremely low number of such cells that we could acquire. Moreover, Tie1 expression was not maintained in vitro. Thus, we could not directly compare the behaviors of Tie1‐positive cells with Tie1‐negative cells from tumor‐bearing mice in terms of their migratory abilities, matrix digestion, sphere formation, and other properties. When we become capable of expanding these Tie1‐positive cancer cells either in vitro or in vivo, their biological significance in malignant progression will be analyzed. In this respect, we showed that sphere‐formation culture enabled cancer cells to maintain a population of Tie1‐positive CSCs in vitro. This finding opens the door to explore cellular functions of the Tie1 protein in CSCs.

## Conflict of Interest

The authors have no conflict of interest.

## Supporting information


**Figure S1.** Flow cytometric analysis of Colon26‐mTie1 cells.
**Figure S2.** Flow cytometric analysis of Tie1 expression in cells derived from one week culture of the Tie1‐positive HT29 population.
**Figure S3.** Gene expression analysis of CD44 and CD133, stem cell markers in Tie1‐positive HT29 cells. Sorted Tie1‐positive and ‐negative cells recovered from transplanted tumors were analyzed by quantitative RT‐PCR.
