# Supplementary figures and images for: Elevated expression of Tie1 is accompanied by acquisition of cancer stemness properties in colorectal cancer
Source: Cancer Med. 2017 May 2;6(6):1378–88. doi: 10.1002/cam4.1072 (PMC5463078; doi:10.1002/cam4.1072)

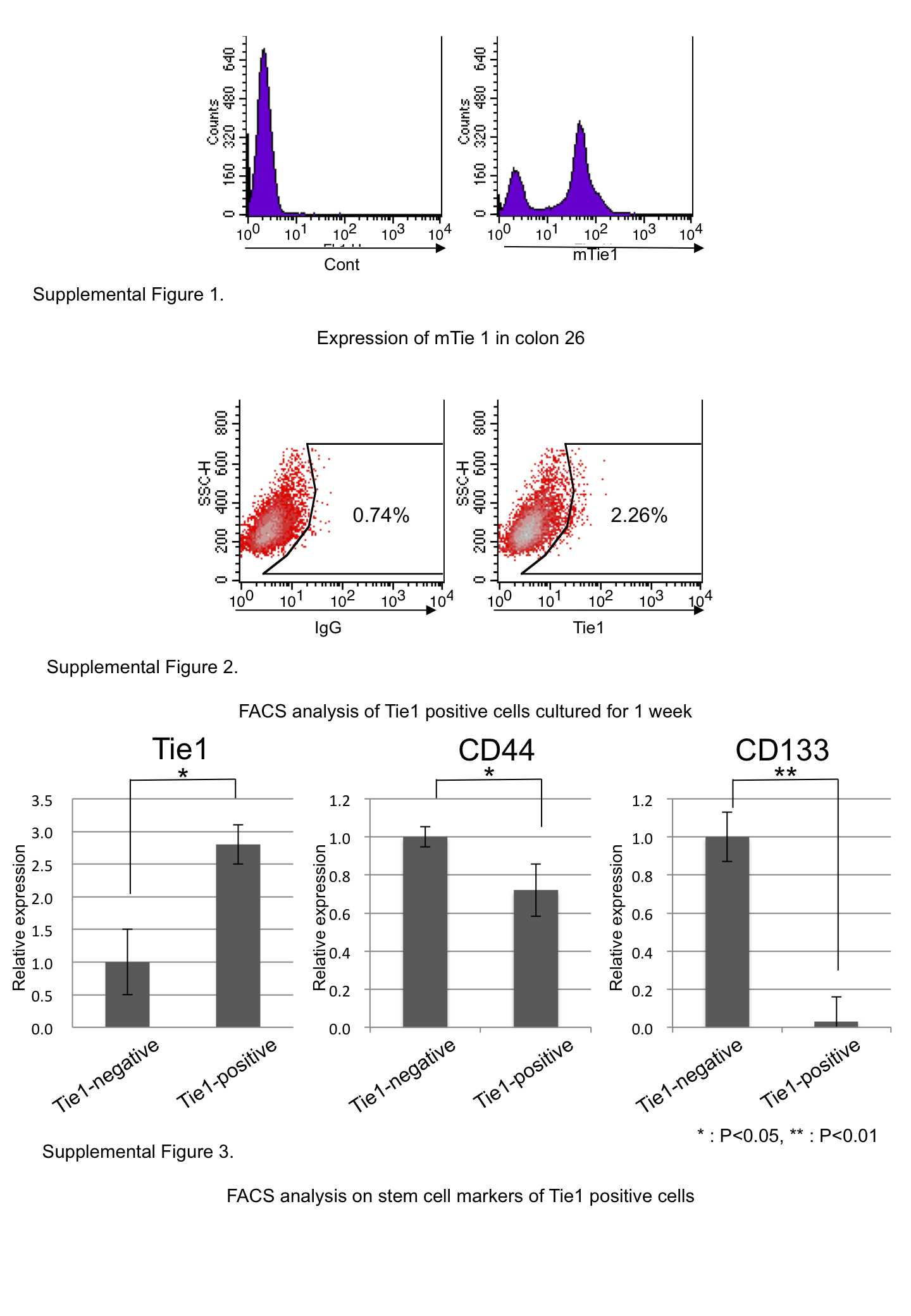

Supplement: Supplementary file 1 — Figure S1. Flow cytometric analysis of Colon26‐mTie1 cells. Figure S2. Flow cytometric analysis of Tie1 expression in cells derived from one week culture of the Tie1‐positive HT29 population. Figure S3. Gene expression analysis of CD44 and CD133, stem cell markers in Tie1‐positive HT29 cells. Sorted Tie1‐positive and ‐negative cells recovered from transplanted tumors were analyzed by quantitative RT‐PCR. [file CAM4-6-1378-s001.tiff]
